# Implementing injury surveillance systems alongside injury prevention programs: evaluation of an online surveillance system in a community setting

**DOI:** 10.1186/s40621-014-0019-y

**Published:** 2014-07-24

**Authors:** Christina L Ekegren, Alex Donaldson, Belinda J Gabbe, Caroline F Finch

**Affiliations:** 1 Department of Epidemiology and Preventive Medicine, Monash University, Alfred Centre, 99 Commercial Rd, Melbourne, 3004 VIC Australia; 2 Australian Centre for Research into Injury in Sport and its Prevention, Federation University Australia, Ballarat, 3353 VIC Australia

**Keywords:** Injury, Surveillance, Safety, RE-AIM framework, Implementation, Qualitative, Interviews, Sport, Australian football

## Abstract

**Background:**

Previous research aimed at improving injury surveillance standards has focused mainly on issues of data quality rather than upon the implementation of surveillance systems. There are numerous settings where injury surveillance is not mandatory and having a better understanding of the barriers to conducting injury surveillance would lead to improved implementation strategies. One such setting is community sport, where a lack of available epidemiological data has impaired efforts to reduce injury. This study aimed to i) evaluate use of an injury surveillance system following delivery of an implementation strategy; and ii) investigate factors influencing the implementation of the system in community sports clubs.

**Methods:**

A total of 78 clubs were targeted for implementation of an online injury surveillance system (approximately 4000 athletes) in five community Australian football leagues concurrently enrolled in a pragmatic trial of an injury prevention program called FootyFirst. System implementation was evaluated quantitatively, using the RE-AIM framework, and qualitatively, via semi-structured interviews with targeted-users.

**Results:**

Across the 78 clubs, there was 69% reach, 44% adoption, 23% implementation and 9% maintenance. Reach and adoption were highest in those leagues receiving concurrent support for the delivery of FootyFirst. Targeted-users identified several barriers and facilitators to implementation including personal (e.g. belief in the importance of injury surveillance), socio-contextual (e.g. understaffing and athlete underreporting) and systems factors (e.g. the time taken to upload injury data into the online system).

**Conclusions:**

The injury surveillance system was implemented and maintained by a small proportion of clubs. Outcomes were best in those leagues receiving concurrent support for the delivery of FootyFirst, suggesting that engagement with personnel at all levels can enhance uptake of surveillance systems. Interview findings suggest that increased uptake could also be achieved by educating club personnel on the importance of recording injuries, developing clearer injury surveillance guidelines, increasing club staffing and better remunerating those who conduct surveillance, as well as offering flexible surveillance systems in a range of accessible formats. By increasing the usage of surveillance systems, data will better represent the target population and increase our understanding of the injury problem, and how to prevent it, in specific settings.

**Electronic supplementary material:**

The online version of this article (doi:10.1186/s40621-014-0019-y) contains supplementary material, which is available to authorized users.

## Background

The development of successful injury prevention strategies is reliant on high-quality epidemiological data about the incidence and severity of injuries (Holder et al. [2001]). In order to be useful for prevention purposes, injury surveillance data should be reliable, valid, representative of the target population and recorded continually over time (Centers for Disease Control and Prevention [2001]). Upholding such standards is a persistent challenge faced by those who implement and maintain injury surveillance systems.

There is now a large body of research aimed at improving standards of practice in injury surveillance (Doraiswamy [1999]; Ezenkwele and Holder [2001]; Orchard et al. [2005]; Fuller et al. [2006]; McKinnon et al. [2009]; Liu et al. [2009]). However, much of this research has focused on issues of data quality rather than upon the implementation of injury surveillance systems (McKinnon et al. [2009]). One of the key reasons for this is that many injury surveillance systems operate within settings where surveillance is mandatory, such as hospitals, where system users are often obligated to conduct surveillance as part of their role (Marson et al. [2005]; Liu et al. [2009]; Doraiswamy [1999]). Hence, there has been less need to focus on ways of encouraging users to adopt and maintain injury surveillance systems.

There are numerous settings where injury surveillance is not mandatory, but its implementation would greatly enhance efforts to reduce injury (Boergerhoff et al. [1999]; Finch and Mitchell [2002]; Finch [2012]; Goode et al. [2014]). One such setting is community sport, where the majority of organised sports participation in Australia takes place (Finch et al. [1999]; Australian Bureau of Statistics [2012]). Sports participation can be associated with numerous injuries and high injury-related healthcare costs (Potter-Forbes and Aisbett [2003]; Tovell et al. [2012]), yet through the delivery of effective injury prevention strategies, many sports injuries are avoidable (Gabbett [2004]; Quarrie et al. [2007]; Emery et al. [2007]; Steffen et al. [2008]; Gilchrist et al. [2008]; Orchard and Seward [2009]; Emery [2010]). To date, it has been difficult to develop effective injury prevention strategies and safety policies for community sports settings as the majority of epidemiological data on sports injuries have been collected on professional and elite athletes, and are not relevant to community-level sporting populations (Finch [2012]).

In order to obtain high-quality epidemiological data on community sports participants, injury surveillance systems are required. However, there are substantial contextual barriers to the implementation of such systems in community sport, including a lack of resources and a reliance on volunteer personnel (Donaldson et al. [2012]). Without mandating injury surveillance in community sports, sports bodies and researchers are faced with the challenge of encouraging club personnel to adopt what is essentially a voluntary task.

A systematic approach is required to understand and overcome the barriers to implementing surveillance systems in this setting. Principles of implementation science are new to the field of injury surveillance research but could potentially enhance these efforts. Very few studies have used theoretical frameworks to guide the development of implementation strategies for surveillance systems (de Mheen PJ et al. [2006]; Zargaran et al. [2014]) and only one surveillance study has incorporated implementation frameworks (such as the RE-AIM framework (Glasgow et al. [1999])) into its evaluation (de Mheen PJ et al. [2006]). As yet, no studies have used principles of implementation science to systematically trial and evaluate the implementation of an injury surveillance system in sport.

This study aimed to i) evaluate use of an online injury surveillance system following delivery of an implementation strategy; and ii) investigate factors influencing the implementation of the system in community sports clubs. To address the first aim, the implementation of the surveillance system was evaluated using the RE-AIM framework. This framework, well-known in the field of implementation science, consists of five domains: reach, efficacy, adoption, implementation and maintenance (Glasgow et al. [1999]). The second aim was achieved via a series of semi-structured interviews conducted with potential end-users of the surveillance system. These investigations were conducted as part of the larger NoGAPS project (National Guidance for Australian Football Partnerships and Safety), a four-year study aiming to prevent injuries via an evidence-informed training program (known as FootyFirst) in community Australian football clubs (Finch et al. [2011]).

## Methods

### Setting and background to the study

Australian football is a popular (Standing Committee on Sport and Recreation [2010]), fast-paced contact sport which involves running and moving the ball by hand (handballing) and foot (kicking) (Australian Football League [2010]). It is associated with numerous injuries (Finch et al. [2013]) and has the highest frequency of hospitalised injuries of any sport in Australia (Flood and Harrison [2006]; Henley [2007]). In 2011, five community Australian football leagues (n = 78 clubs, approximately 4000 athletes) in the state of Victoria, Australia agreed to be involved in the parent project. For the purposes of this project, the five leagues were allocated to one of three study arms, each receiving a different level of support for the delivery of FootyFirst (Finch et al. [2011]). Arm 1 consisted of two regional leagues (n = 22 clubs) in South-Western Victoria; arm 2 consisted of one large metropolitan league (n = 31 clubs); and arm 3 consisted of two regional leagues in North-Western Victoria (n = 25 clubs). The FootyFirst program, designed to be delivered by an Australian football coach, includes a combination of dynamic stretches, strengthening exercises, and jumping/landing techniques. It is targeted at preventing ankle, knee, hamstring, groin and hip injuries in community Australian football players (Donaldson [2014]).

To better understand the implementation context for injury surveillance activities and improve the design of our implementation strategy, we asked the leagues’ Chief Executive Officers (CEOs) about the feasibility of ongoing injury surveillance within their leagues. No league had a mandatory injury surveillance policy in place, but all CEOs expressed an interest in introducing one. Where surveillance was used, club personnel (e.g. sports trainers) used various non-standardised methods to record injuries, mainly for their own purposes (personal communications, 18 November, 2011).

To further our understanding about injury surveillance activities within clubs, we then conducted a pre-implementation survey of sports trainers from participating clubs within the five leagues (Ekegren et al. [2012]). Sports trainers are non-medically trained personnel employed by sports clubs to provide first-aid and injury management. In summary, 87% of the 33 respondents (32% response rate) recorded injuries at their club on a routine basis, mostly using paper-based notebooks or forms. Amongst respondents, attitudes towards injury surveillance were positive and ‘sports trainers’ were identified as those who should be primarily responsible for recording injuries at clubs.

### Participants and recruitment

When designing an implementation strategy for any action, the first of several core implementation components to be considered is staff selection (Fixsen et al. [2009]). League CEOs and sports trainers were in agreement that sports trainers were the most appropriate staff for conducting injury surveillance. In Australian football, sports trainers provide on-site first aid at some training sessions and all matches, referral to external medical or allied health experts if necessary, and ongoing injury management (Zazryn et al. [2004]; Casey et al. [2004]). Sports trainers may not have healthcare backgrounds but, in Australian football, they must all complete an endorsed first aid and athlete safety training course (Donaldson and Finch [2012]).

Before the start of the 2012 football season, league CEOs invited those sports trainers whose email addresses they held to attend an information session on the proposed injury surveillance system. For many clubs, the league did not have sports trainers’ email addresses, so instead they contacted the club’s coach and asked them to pass on an invitation to their trainer(s). Information session attendees provided their contact details to the research team to enable follow-up regarding the injury surveillance system. Sports trainers who did not attend information sessions were contacted individually by phone and/or email (via their club’s coach) about participating in the injury surveillance project. These recruitment procedures were repeated at the start of the 2013 season to capture any clubs not recruited in 2012 or who had changed their sports trainers between seasons. Monash University Human Research Ethics Committee granted ethics approval for all procedures.

### Procedures

The information sessions were part of a multifaceted implementation strategy designed to maximise uptake of the system across the three study arms (described later). The strategy incorporated several core implementation components, including training, ongoing coaching and consultation, and performance evaluation (Fixsen et al. [2009]). The injury surveillance system implementation strategy was carried out before and during the 2012 and 2013 seasons and consisted of three main elements:

1. Information sessions. The research team conducted information sessions at each league headquarters for sports trainers or other club personnel interested in the proposed injury surveillance system. These sessions focused on raising awareness of the value of injury surveillance, including how to use surveillance data to design and evaluate injury prevention strategies. An online surveillance tool was also demonstrated to the attendees. In two out of the three sessions, our presentation was incorporated within a package of presentations to sports trainers (e.g. updates on practice guidelines or instructions on taping).
2. Personal instruction. Each information session attendee was contacted by phone, email or personal visit and provided with further instructions about setting up their online surveillance account. They were sent a user manual and documentation for them and their coaches to sign, enrolling their club in the project. Users were also provided with the primary author’s (CLE) email address so that they could request personalised technical support as required. They were asked to provide a mobile phone number and agree to receive weekly short message service (SMS) reminders about recording injuries throughout the season.
3. Weekly reminders. The primary author (CLE) logged onto the online system each week during the season to review who had recorded injuries that week. An SMS reminder (including a request to inform us if there had been no new injuries) was sent to those who had not recorded any injuries. A thank you message was sent to those who had recorded injuries.

### Online surveillance tool and surveillance procedures

**Figure 1:**
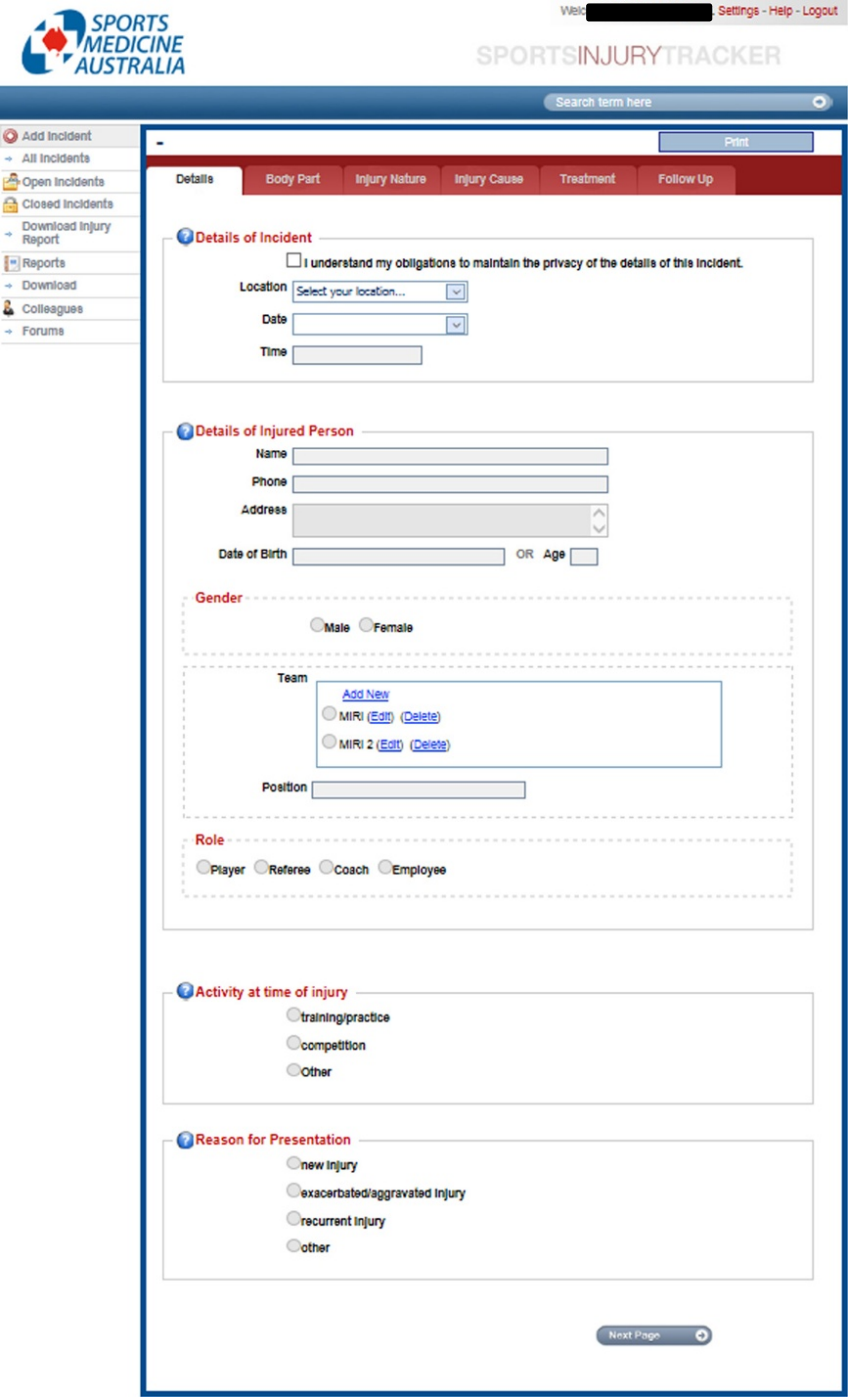
**Screenshot of the first page of six to be completed for each injury entered into the online surveillance tool.**

The Victorian branch of Sports Medicine Australia (SMA), Australia’s major sports medicine advisory body, developed Sports Injury Tracker as an online tool for recording information about specific injury events. Users click through six pages completing a range of data fields (Figure 1) by selecting from a list of response options or providing free-text responses where appropriate. The injury variables to be recorded in the online tool are as follows (Sports Medicine Australia 2012):

1. Date of injury
2. Type of activity at time of injury (e.g. match/training)
3. Reason for presentation (e.g. new/recurrent/exacerbated injury)
4. Mechanism of injury (e.g. struck by other player/etc.)
5. Body region injured (e.g. shoulder/thigh/ etc.)
6. Nature of injury (e.g. abrasion/ fracture/etc.)
7. Initial treatment (e.g. none/ crutches/ etc.)
8. Action taken (e.g. immediate return/etc.)
9. Referral (e.g. no referral/ physio/etc.)
10. Provisional severity assessment (mild/moderate/severe)
11. Treating person (e.g. Medical practitioner/etc.)
12. Return to football date

Once an injury event is recorded, a page is created summarising the injury. Graphs and spreadsheets summarising recorded injuries can be downloaded. A paper-based version of Sports Injury Tracker system is also available, allowing recording and transfer to the online system at a later date [see Additional file 1].

As part of their personal instructions, sports trainers were asked to record ‘any new football-related injury occurring during football training sessions or matches’ including overuse and traumatic injuries. They were asked to do this every week, recording any new injuries occurring in the previous seven days. Before the start of each football season, participating sports trainers informed all athletes at their clubs about the study and gave them an opportunity to ask questions. Athletes who did not want their injury details recorded could opt-out, but only one individual chose this option.

### Evaluation

The evaluation consisted of two parts — a quantitative evaluation using the RE-AIM framework (Glasgow et al. [1999]) and qualitative semi-structured interviews exploring factors affecting implementation of the injury surveillance system.

#### Quantitative evaluation

The RE-AIM framework, widely used in implementation science, consists of five domains: reach, efficacy, adoption, implementation and maintenance (Glasgow et al. [1999]). As RE-AIM was originally designed to evaluate the public health impact of interventions (Glasgow et al. [1999]), we re-operationalised the five domains in order to apply them to the implementation of an injury surveillance system (Table 1). For this study we defined ‘reach’ as the proportion of the target population (representatives from 78 clubs) who attended an information session about the surveillance system or had phone/email contact with a research team member expressing interest in using the system. The term ‘efficacy’ is not often applied to injury surveillance systems. Instead, terms conveying the quality of the recorded data, such as ‘validity’ or ‘completeness’ are used to indicate that a surveillance system is operating successfully (Centers for Disease Control and Prevention [2001]). The quality of data recorded by sports trainers using the online tool has been previously reported and readers are referred to this publication for further details about the ‘E’ domain of the RE-AIM framework in the context of this study (Ekegren et al. [2014]. doi:10.1111/sms.12216.). ‘Adoption’ was defined as the proportion of football clubs that agreed to participate and set up an online account with the intention of conducting injury surveillance. In relation to the ‘implementation’ of the surveillance system, we did not consider clubs to have fully implemented the system if they recorded less than 10 injuries per football season. Previous research about the frequency of injury in community Australian football (Finch et al. [2013]) would suggest that such low injury numbers in a standard club of 50 players would be a significant underestimate and would indicate that surveillance had not been conducted with adequate diligence. Finally, ‘maintenance’ was defined as the proportion of football clubs implementing the surveillance system in 2013, after previously doing so in 2012.

**Table 1:** **RE-AIM domain definitions—original and re-operationalised for implementation of an injury surveillance system**

Domain	Original definition (Glasgow et al. [1999] )	Definition as applied to an injury surveillance system
**R** each	Proportion of the target population that participated in the intervention	Proportion of football clubs informed about and/or trained in use of the injury surveillance system
**E** fficacy	Success rate if implemented as in guidelines	Data quality (see (Ekegren et al. [2014]. doi:10.1111/sms.12216.)
**A** doption	Proportion of settings, practices, and plans that will adopt this intervention	Proportion of football clubs that agreed to participate and set up a Sports Injury Tracker account with the intention of conducting injury surveillance
**I** mplementation	Extent to which the intervention is implemented as intended in the real world	The proportion of football clubs recording injuries using Sorts Injury Tracker throughout season (not including clubs recording <10 injuries throughout season)
**M** aintenance	Extent to which a program is sustained over time	The proportion of football clubs implementing the surveillance system in 2013 after doing so in 2012.

The surveillance system implementation strategies were delivered equally across the three study arms over both study years. However, there were differences between the study arms in the level of support provided by researchers for the delivery of FootyFirst. Arm 1 received FootyFirst with full delivery support over both years. Arm 2 acted as the control arm in Year 1 and received FootyFirst (with full delivery support) only in Year 2. Arm 3 received FootyFirst with minimal delivery support over the two years (Finch et al. [2011]). It was hypothesised that aspects of this support, such as club engagement, asking for clubs’ input into the project and assigning FootyFirst mentors to participating clubs, could lead to a greater compliance with all aspects of the project, including the injury surveillance component. Therefore, RE-AIM domains were analysed separately for each arm of the parent project. Descriptive statistics were used to evaluate system reach, adoption, implementation and maintenance.

#### Qualitative evaluation of factors affecting implementation of injury surveillance system

At the end of the 2012 football season, individuals who had initially been ‘reached’ by the intervention in 2012 (n = 37) were contacted in random order and invited to participate in follow-up interviews about the injury surveillance system. To gauge a diverse range of opinions purposive sampling was used to ensure even-capture of individuals who had and had not implemented the system in 2012 (Barbour [2001]). The primary author conducted and audio-recorded 30–60 minute semi-structured, face-to-face or phone interviews using a standardised interview guide. Recruitment and interviewing continued until the primary author considered that content saturation was reached within both groups (Green and Thorogood [2009]).

The interview guide was developed based on a previous survey carried out during the 2012 pre-season (Ekegren et al. [2012]). It included a range of open-ended questions about factors influencing the implementation of the surveillance system, as well as past and current injury recording practices and questions about the online surveillance tool. Examples of interviewer prompts are shown in Table 2.

**Table 2:** **Examples of interviewer prompts used in semi-structured interviews**

**Injury surveillance practices – i.e. What do you do?**	Did you have a previous system in place for monitoring injuries at your club? Please describe it.
	On average, how much time do you spend each week recording injuries?
	Do you intend to conduct injury surveillance next season?
**Factors influencing injury surveillance practices – i.e. Why do you do it?**	What were your main reasons for carrying out injury surveillance this season?
	Within your football club, who should be primarily responsible for recording injuries?
	Would it be helpful to be provided with more training or support on how to record injuries? Who should provide this?
	What kind of information would you like to be able to produce from your injury data? What would you use it for?
	What has been the club’s/coach’s attitude towards you carrying out injury surveillance?
	Could you suggest any ways to make it easier to record injuries at your club?
**Specific questions about online surveillance tool**	How did you first find out about Sports Injury Tracker?
	What is your opinion on using an online tool to record injuries?
	Have there been any difficulties accessing a computer or the internet in order to use the online system?
	Would you want to modify or adapt the system in any way?

Audio-recordings of the interviews were transcribed and verified by interviewees before being thematically analysed using open-coding to identify key themes (Hsieh and Shannon [2005]). NVivo Version 10 (QSR International Pty Ltd, 2012) was used to assist with data analysis. Three interviews from each group of interviewees were randomly selected and independently coded by the primary author and a research assistant to develop a common coding framework consisting of fewer, higher-level themes to be used for all subsequent coding. All interviews were then coded by the primary author using this coding framework. Six interviews were double-coded (by the primary author and a research assistant) to enable cross-checking of data interpretation (Barbour [2001]). Where discrepancies arose, these were discussed and, where necessary, themes modified further.

## Results

### RE-AIM evaluation

**Figure 2:**
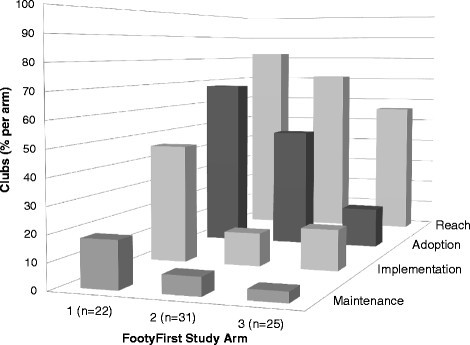
**Reach, adoption, implementation and maintenance of injury surveillance system over two years.** Results are displayed as the percentage of total clubs per FootyFirst study arm.

The results of the RE-AIM evaluation are shown in Table 3 and Figure 2. Each of the five domains is discussed below.

**Table 3:** Reach, adoption, implementation and maintenance (R(E)*-AIM evaluation) of online injury surveillance system over two years

Study Arm	Study year	Reach	Adoption	Implementation	Maintenance
		n (%)	n (%)	n (%)	n (%)
**1** (n = 22) (Received full delivery support for FootyFirst in years 1 and 2)	1	15 (68%)	12 (55%)	7 (32%)	n/a
	2	11 (50%)	8 (37%)	7 (32%)	**4 (18%)**
	**Both**	**18 (82%)**	**15 (68%)**	**10 (46%)**	n/a
**2** (n = 31) (Received full delivery support for FootyFirst in year 2 only)	1	10 (32%)	7 (23%)	5 (16%)	n/a
	2	19 (61%)	11 (36%)	2 (7%)	**2 (7%)**
	**Both**	**22 (71%)**	**15 (48%)**	**4 (13%)**	n/a
**3** (n = 25) (Received minimal delivery support for FootyFirst in years 1 and 2)	1	12 (48%)	3 (12%)	3 (12%)	n/a
	2	6 (24%)	2 (8%)	2 (8%)	**1 (4%)**
	**Both**	**14 (56%)**	**4 (16%)**	**4 (16%)**	n/a
Total (n = 78)	1	37 (47%)	22 (28%)	15 (19%)	n/a
	2	36 (46%)	21 (27%)	11 (17%)	**7 (9%)**
	**Both**	**54 (69%)**	**34 (44%)**	**18 (23%)**	n/a

NB: Maintenance was always n/a for study year 1 (2012), because it was defined as the proportion of clubs that implemented the system in 2013, after already doing so in 2012.

*NB. Readers are referred to Ekegren et al. [2014]. doi: 10.1111/sms.12216 for the results of the evaluation of the ‘E’ domain of the RE-AIM framework.

Results are displayed as n clubs and percentage of total clubs per FootyFirst study arm.

#### Reach

Fifty four (69%) of the 78 clubs eligible across the five leagues were reached by the injury surveillance implementation strategy over two years. We reached 37 clubs (47%) in 2012 and an additional 17 clubs (23%) in 2013. There were various reasons for why we did not reach the remaining 24 clubs—three clubs refused to participate from the outset, the sports trainer(s) from six clubs did not respond to requests for information, and we were unable to obtain the sports trainers’ details for 15 clubs. We reached the greatest proportion of clubs (82%) in arm 1 and the lowest proportion in arm 3 (56%). For arms 1 and 3, reach was higher in 2012 compared to 2013 and for arm 2, reach was higher in 2013 (Table 3).

#### Efficacy

In our previously published study on the quality of the injury surveillance data, we reported a range of data quality variables, including a) the proportion of injuries captured by the surveillance system compared to self-report by athletes; b) the completeness of the data recorded in the surveillance system; and c) the agreement between the profiles of injury data recorded using the surveillance system and athlete self-report (Ekegren et al. [2014]. doi:10.1111/sms.12216.). Readers are referred to that study for full results but to summarise, we found that the profile of injuries reported by sports trainers was consistent with previous studies and there was a high level of completeness of injury records. However, we also found significant variability across clubs in the injury reporting rate with some clubs greatly underreporting the frequency of injuries.

#### Adoption

Thirty-four (63% of the 54 clubs reached and 44% of all 78 clubs) clubs adopted the surveillance system as measured by agreement to participate in the study and by setting up an online surveillance account. Again, we achieved the highest level of adoption among clubs in arm 1 (68%) and the lowest in arm 3 (16%). For arms 1 and 3, adoption was higher in 2012 compared to 2013 and for arm 2, adoption was higher in 2013 (Table 3).

#### Implementation

Eighteen clubs (53% of the 34 clubs that adopted the surveillance system and 23% of all 78 clubs) fully implemented the system by recording ten or more injuries using the online tool. A further five clubs recorded fewer than ten injuries over the season and these clubs were excluded from analyses. The highest level of implementation of the surveillance system was achieved in arm 1 (46% of all clubs) and arm 2 demonstrated the lowest level of implementation (13%). For arm 1, the level of implementation was maintained from 2012 to 2013 but for arms 2 and 3, implementation declined over the two years (Table 3).

#### Maintenance

Seven clubs (47% of the 15 clubs who implemented the system in 2012 and 9% of all 78 clubs) continued to implement the system in 2013. Arm 1 demonstrated the highest level of maintenance (18% of all clubs, n = 4) and arm 3 the lowest (4%, n = 1). The eight clubs that discontinued using the system gave a range of reasons including: the people responsible for surveillance left the club and no one was willing to take over from them (n = 4), technical issues with the system leading to giving up on the system (n = 1), and reverting to a previous injury recording system in a notebook because the new system was too complex for their needs (n = 1). Two clubs did not give any reasons for discontinuing.

### Qualitative evaluation

#### Profile of interviewees

Twelve individuals were interviewed before content saturation was achieved. All six interviewees who had implemented the injury surveillance system and four of the six who had not implemented the system were sports trainers; the remaining interviewees were a football manager and a head coach who had opted to do the injury recording themselves. The interviewees had completed training relevant to their roles and some also had additional professional training (e.g. physiotherapy, osteopathy, nursing, massage and emergency medical services). There was an even representation of males and females (Table 4). Most interviewees were aged 30–49 years and the majority had 2–10 years of experience in their current role. Of the interviewees who had implemented the injury surveillance system, four were new to their role at the club and had not conducted any injury recording previously (Table 4).

**Table 4:** **Demographic characteristics of interviewees who did/did not implement the online injury surveillance system**

Characteristic	Implemented surveillance system	Did not implement surveillance system
	(n)	(n)
Role at club		
Sports trainer	6	4
Other	0	2
Sex		
Female	2	3
Male	4	3
Age group		
18–29 years	1	1
30–49 years	3	4
50 + years	2	1
Time in current role		
Less than 2 years	2	1
2 to 10 years	4	5
More than 10 years	0	0
Previous method of injury recording		
Sports Injury Tracker	-	-
Other paper-based form/notebook	1	3
Computer spread sheet	1	1
No injury recording	-	2
New to club	4	-
**Total**	**6**	**6**

#### Level of implementation amongst interviewees

Of the 12 interviewees, 6 fully implemented the online surveillance system. Out of the six non-implementers, five adopted the intervention (i.e. opened a Sports Injury Tracker account) but did not record any injuries. The remaining non-implementer was reached by the intervention (i.e. knew about the system) but did not open an account, reportedly due to a lack of time. These six non-implementers had either retained their previous injury recording methods (computer spread sheets (n = 1) and notebooks (n = 3)) or were not recording injuries at all (n = 2). Where notebooks were used, interviewees reported filling these out inconsistently, with many injuries going unrecorded.

#### Factors influencing surveillance system implementation

A range of factors influencing interviewees’ implementation of the surveillance system were identified. Three main themes emerged from the data: i) factors that influenced the individual responsible for conducting injury surveillance (‘personal factors’); ii) factors relating to social connections within football clubs and to the culture of community Australian football (‘socio-contextual factors’); and iii) factors relating to the online surveillance tool itself (‘system factors’). Within these three main themes, key findings emerged as both facilitators and barriers to implementing the injury surveillance system. These are summarised in Table 5 and discussed in detail below.

**Table 5:** **Factors influencing injury surveillance practices: themes and supporting quotes**

Themes and key facilitators and barriers	Supporting quotes
**1. Personal factors**	
Facilitators	*‘I mean, looking after upwards of sort of 70 people it’s sort of hard to keep track of a lot of the injuries… and if that person has followed up with the advice that you’d given them or if they went and got the referral. And so, it was really to just sort of check up with people. Which is yeah, really handy. Sort of … keeping track of everyone.’*
Belief in the importance of injury surveillance	
Injury surveillance as part of sports trainer’s role	*‘Oh look, I suppose I’m a little bit different to a traditional football trainer. I probably don’t meet the mould…. And I’m just sort of hungry for those types of processes. Where I can monitor players and I understand that you know, it does work.’*
Barriers	
Lack of importance placed on injury surveillance	*‘I mean, we know who the people with the repeat offenders are anyway. You don’t really need a stat saying that.’*
**2. Socio-contextual factors**	
Facilitators	
Association with FootyFirst	*‘So, we were trying to use them [the surveillance system and the training program]… working in conjunction with each other so we could sort of see the benefits of the FootyFirst programme.’*
Barriers	
Lack of/transience of staff	*‘Look, it was probably not enough. It was really all that we had. With the resources that we had available. And staff. Sort of up until this year and last year we really haven’t had the specialists on board … So, we’ve sort of had a limited knowledge base that we can work with.’*
Underreporting of injuries	*‘There’s a photo on Facebook. Soccer players pretend to be hurt, football players pretend they’re not.’*
Lack of support/leadership	*‘Yeah, I think that would get more clubs involved as well. If [the league] sort of pushed it a bit more. And even talking to the actual… the clubs over a whole. So, coaching staff and everything. As well as the trainers. So, that everyone’s aware of it. And everyone will sort of talk about it more.’*
	*‘Because I mean, if you’ve got the support of the league and SMA and the club then usually there’s no problem.’*
**3. System factors**	
Facilitators	
Ease of use	*‘Well, like I said I’m not the greatest on computers and I seem to… I got through it. So, I think if I can get through it you’ve pretty much got… you’ll cover most people.’*
Barriers	
Time taken to upload injuries	*‘It takes two seconds to write it down with a pen. It took about five minutes to enter one injury in. … and when you have… you know, when you have ten or so injuries to record. Yeah, it took a while.’*
Technical issues	*‘And then yeah, after that one time when it didn’t really work for I don’t know why it wouldn’t. But it wasn’t really saving data at all. So, after that I did keep it in a book for probably about four or five weeks.’*
Data requirements	*‘You should probably try and [cut] it down so not so many pages have to be clicked through….Because you do have to go through a few pages. And other things that you’ve almost got to wait for the feedback to be able to put that information in properly. You know, you’re not sure if he’s torn that ligament.’*
Adjusting to a new system	*‘I’m happy to do that paperwork because generally when I do my notes, it’s during the game…. But then afterwards yeah, it’s finding that time ….I suppose that probably just didn’t work for me because I was new at it and I hadn’t mastered the system yet.’*

### 1a. Personal factors facilitating injury surveillance

#### Belief in the importance of injury surveillance

Several interviewees indicated that a belief in the importance of injury surveillance was a strong motivator for them to engage in the practice. Interviewees gave a range of reasons for the importance of injury surveillance, including: i) to act as a memory aid; ii) to facilitate communication between club personnel (e.g. within the first aid/medical team or between trainers and coaches); iii) for legal reasons (e.g. advice given to athletes following injury); and iv) to try to determine injury causation and develop injury prevention strategies. Where clubs had implemented the injury surveillance system, it was maintained only through the diligence of the individual responsible. It was apparent that those interviewees who had implemented the system had a personal interest in the process and were intrinsically motivated to continue.

#### Injury surveillance part of sports trainer’s role

Interviewees who had implemented the surveillance system generally felt responsible for injury surveillance at their club. They acknowledged that recording injuries took extra time and was beyond their normal duties yet they did not consider this unreasonable, with one interviewee stating ‘two to four hours a week for me would be nothing’.

### 1b. Personal factors acting as a barrier to injury surveillance

#### Lack of importance placed on injury surveillance

Two interviewees who did not record injuries considered the formal process of injury surveillance to be unimportant because they believed they could remember all of their athletes’ injuries without writing them down. One also felt that club-based record keeping was unnecessary, as injuries were recorded by the treating physiotherapist external to the club. Another interviewee suggested that clubs within their league did not want to conduct surveillance because such practices would be seen as ‘taking it all a bit too seriously’.

### 2a. Socio-contextual factors facilitating injury surveillance

#### Association with FootyFirst

In most cases, the interviewee was initially told about and encouraged to use the injury surveillance system by their club’s coach or president (who had heard about the system from the research team). Coaches who decided to be involved with the FootyFirst program also encouraged their trainers to sign up to use the surveillance system. Five of the six implementers, but only two of the six non-implementers interviewed were from clubs that had agreed to be involved with FootyFirst.

### 2b. Socio-contextual factors acting as a barrier to injury surveillance

#### Lack of/transience of staff

For most non-implementers, a lack of staff was cited as a key barrier to conducting injury surveillance. Several clubs only had two sports trainers on staff and they struggled to keep up with the usual demands of their role without engaging in extra duties. One interviewee felt that an absence of ‘specialist’ staff at their club (e.g. physiotherapists) precluded them from recording injuries and implied that sports trainers were not suitable for the task.

Before the start of the following football season, 4 of the 12 interviewees resigned from their roles at the clubs. Two doubted whether anyone else would continue recording injuries in their absence. This transience of staff may be due to the working conditions of sports trainers, with several interviewees commenting that being a sports trainer was almost like being a volunteer, i.e. not highly paid, and done for the experience or love of the sport.

#### Underreporting of injuries

The majority of interviewees raised the issue of athlete underreporting of injuries as a barrier to injury surveillance. Some reported that athletes did not want to reveal their injuries in case this resulted in them missing a match. This desire to play when injured was compounded by the attitudes of some coaches who also encouraged athletes to play when injured. It was also reported that athletes often ignored their injuries, refusing to seek help from trainers or other health professionals and continuing to play. The reasons for this include athletes being unable or unwilling to pay for treatment by external health professionals, or athletes not considering themselves injured.

#### Lack of support/leadership

While many coaches had initially encouraged trainers to use the injury surveillance system, most interviewees reported that coaches and club administrators were not interested in the specific injury surveillance outcomes. Even when interviewees had analysed injury data to create end-of-season summary reports, they did it for personal interest rather than for anyone else at the club. For the most part, sports trainers felt that coaches were interested in ascertaining who could play each week, not using the data for injury prevention purposes.

Regardless of whether they had implemented the surveillance system or not, interviewees felt that more support from their coach, club, and league would have helped to facilitate ongoing injury surveillance. When asked whether, at the conclusion of the project, there should be a body responsible for carrying on the administration and support for the online system and if so, who this should be, many interviewees felt that the peak sports medicine authority, SMA, should fulfil this role.

### 3a. System factors facilitating injury surveillance

#### Ease of use

Several interviewees commented that, although they had limited computer skills, they were able to use the system. When asked whether the training they received was adequate or if they would have found the system easier to use with more thorough training, most interviewees (including those who did not implement the system) thought the system was self-explanatory and no more training was necessary. However, it was universally acknowledged that individuals who were completely unfamiliar with computers would probably struggle to use the online system.

### 3b. System factors acting as a barrier to injury surveillance

#### Time taken to upload injuries

Most interviewees acknowledged that entering information into the system increased the time and the effort required to record injuries. This was the major barrier cited by non-implementers. Several interviewees recorded injuries on the paper-based version of the form or in a notebook during training and matches, and then uploaded the data onto the system later. The time spent doing this ranged from 15 minutes to 2 hours per week. To streamline the process, some interviewees tried recording injuries using their smart phones as soon as they occurred. However, there was no mobile version or mobile application (‘app’) for the online tool and it was difficult to use the system on such devices. Several interviewees suggested that an app should be developed.

How interviewees interpreted the definition of a recordable injury also influenced the amount of time spent recording injuries. Some interviewees recorded all injuries including lacerations and bruises while others only recorded more serious injuries leading to an athlete missing a match. Many interviewees agreed that a narrower injury definition would reduce the amount of time they spent recording injuries.

#### Technical issues

Three interviewees commented on technological issues they had experienced with the system including the length of time it took to load, leading to it taking too long to record each injury. Also, sometimes injury details did not save properly and had to be re-entered. One user also tried to link several colleagues within their club so that they could all log in and view the system. However, the process was quite complicated and there were no instructions in the user manual to guide this. There were other issues raised concerning the reporting functions and the ability to filter injuries based on teams.

#### Data fields

Some interviewees felt that too much detail was required for each injury and that certain information, such as the injury diagnosis, was often unavailable at the time of injury. Others were not satisfied with the response options for certain data fields. In particular, three interviewees mentioned that the options for the mechanism of injury could be more detailed. One interviewee also commented that the paper-based form differed to the online version which added to the challenge of transferring data at a later date.

#### Adjusting to a new system

Four of the six non-implementers continued using their pre-existing injury recording system rather than changing to the new online system. Most of these individuals recorded injuries in notebooks, preferring the immediacy and simplicity of this format compared to the online system. Conversely, four of the six implementers were new to their role at the club and therefore, were happy to try the new system in the absence of a pre-existing alternative.

## Discussion

We aimed to evaluate the use of an injury surveillance system in a community sports setting and describe relevant barriers and facilitators. For injury surveillance data to be useful for injury prevention purposes it must be both high-quality and representative of a large proportion of the target population (Centers for Disease Control and Prevention [2001]). This study was unique in that most previous research on injury surveillance systems has focused on factors influencing data quality rather than on factors influencing the implementation of the system (McKinnon et al. [2009]). In sport, there have been no studies that have used principles of implementation science to systematically trial and evaluate the implementation of an injury surveillance system.

We evaluated the reach, adoption, implementation and maintenance of the injury surveillance system over two consecutive football seasons. In a previous study, we also evaluated system efficacy over a single season (Ekegren et al. [2014]. doi:10.1111/sms.12216.). In relation to the four RE-AIM domains measured in the present study, the proportions of clubs achieving reach (69%) and adoption (44%) were a reasonable representation of the 78 clubs targeted. However, the proportions of clubs implementing (23%) and maintaining the system (9%) were low. Consistent with key implementation-science concepts, the implementation strategy was developed following consultation with multiple tiers of influence, including league administrators and system-users, and was based on several core implementation components (Finch and Donaldson [2010]; Fixsen et al. [2009]). However, the poor outcomes achieved in the implementation and maintenance of the injury surveillance system suggests that our implementation strategy was inadequate or inappropriate for this setting.

Although the implementation strategy for the surveillance system was the same for all study arms, there were marked differences in the RE-AIM outcomes between the arms (Table 3). These differences appear to relate to the level of support provided for the delivery of FootyFirst (Finch et al. [2011]). Within leagues receiving full delivery support for FootyFirst (study arm 1 (in 2012 and 2013) and study arm 2 (in 2013)), there was a high profile launch for the project and extensive personal contact between the research team and club personnel, including coaches, presidents and sports trainers. There was also encouragement from the league to participate in FootyFirst and information provided to clubs about it. These actions may have helped to endorse the research team and the entire project, including the surveillance system. Results from the qualitative evaluation supported this, with interviewees perceiving a close link between the injury surveillance system and the injury prevention program. Also, coaches who had decided to implement FootyFirst often encouraged their sports trainers to engage with the project, which would have influenced the reach and adoption of the system. In 2013, when full support for FootyFirst was provided to study arm 2, reach and adoption increased. However, the level of implementation decreased. Therefore, it is likely that the support for the delivery of FootyFirst influenced the reach and adoption of the surveillance system, but not its implementation. Other potential influences on implementation were explored further via the qualitative evaluation.

Because injury surveillance is optional in community sport, personal factors impacting on implementation of the surveillance system were common. For example, a belief in the importance of surveillance was a key theme. Recognising injury surveillance as part of the sports trainer’s role influenced implementation and the amount of time interviewees were willing to spend recording injuries. It was evident that a passion for the job motivated several interviewees to engage in these extra duties outside of normal working hours. Other studies evaluating user experiences of injury surveillance systems have reported that outcomes are enhanced when users have accountability for recording injury data (Ezenkwele and Holder [2001]; Porcheret et al. [2004]; Doraiswamy [1999]; de Mheen PJ et al. [2006]). For example, a Dutch hospital-based study highlighted a lack of accountability as a key barrier to the implementation of an adverse-outcome reporting system (de Mheen PJ et al. [2006]). They suggested that, with voluntary reporting systems, offering financial or additional human resource incentives can increase engagement or else the system is reliant solely on the motivation of individuals keen to improve the quality of practice (de Mheen PJ et al. [2006]).

Our study identified important socio-contextual factors influencing the implementation of the injury surveillance system. The most commonly reported contextual barrier was the underreporting of injuries by football players to avoid missing matches. An attitude of invincibility amongst players was also reported, such that injuries were possibly seen as a sign of weakness. Injury underreporting has been recognised in other contexts where physical toughness is valued (e.g. in the defence forces) (McKinnon et al. [2009]). Other important barriers included inadequate staffing levels and a lack of support for ongoing surveillance from leaders and administrators. The influence of peer leadership and social support has been well documented in relation to the use of surveillance systems (de Mheen PJ et al. [2006]; Ezenkwele and Holder [2001]; Gambel et al. [1999]; Spaite et al. [1990]; Boergerhoff et al. [1999]; Finch and Mitchell [2002]).

These findings of the importance of socio-contextual factors are accordant with Fixsen et al’s core implementation components (Fixsen et al. [2005]), i.e. ‘the most essential and indispensable components of an implementation practice or program’ (Fixsen et al. [2005]). The core components can be categorised as those addressing the competency of the individual, organisational factors and leadership issues (Donaldson and Finch [2013]). The implication is that it is insufficient to provide training and coaching to individual practitioners without also targeting those in leadership roles and addressing organisational and cultural barriers. The findings of this study suggest that our implementation strategies focused too heavily on the competency of those charged with implementing the surveillance system (e.g. staff selection, training, coaching and evaluation) without adequately addressing organisational issues (e.g. system interventions and administration) or leadership drivers (e.g. role of senior coach and other club leaders) (Fixsen et al. [2005]; Donaldson and Finch [2013]). Uptake was highest in the FootyFirst study arms receiving maximum program delivery support across multiple tiers of influence, including league administrators, coaches and club presidents, highlighting the importance of organisational and leadership drivers (Finch and Donaldson [2010]).

Interviewees’ perceptions of the online surveillance tool indicated that some were frustrated by the extra step involved in uploading paper records (filled out at the time of injury) to the online tool at a later time. As suggested by previous studies (Goode et al. [2014]), a smartphone or tablet ‘app’ would help address these concerns. Fixing the technical glitches with the system experienced by some interviewees would also encourage more users to persevere with the system. These two barriers are supported by the Diffusion of Innovations construct of ‘complexity’ which suggests ‘the complexity of an innovation, as perceived by members of a social system, is negatively related to its rate of adoption’ (Rogers [2003]). In other words, the more difficult an injury surveillance system is perceived or experienced to be, the fewer the number of people who will be prepared to adopt and implement it. The need for simplicity and utility is well supported by previous literature on surveillance systems (Goode et al. [2014]; Finch et al. [1999]; McKinnon et al. [2009]; Zargaran et al. [2014]).

Another commonly reported barrier was the difficulty changing to a new system when there was a pre-existing system in place. This barrier has been reported previously in surveillance research (de Mheen PJ et al. [2006]; Finch et al. [1999]) and is also consistent with the Diffusion of Innovations construct of ‘compatibility’ which is ‘the degree to which an innovation is perceived as consistent with existing values, past experiences and needs of potential adopters’ (Rogers [2003]). Hence, there may be a need for a more flexible approach to surveillance in community sport such as continuing to provide standardised paper-based reporting forms rather than expecting all clubs to implement computerised online systems. This preference for dual reporting methods has been raised previously in other settings (Goode et al. [2014]; McKinnon et al. [2009]). Our results also suggest that new staff members may be less resistant to using a new surveillance system and therefore, their arrival at a club may present an opportunity for a change in injury surveillance practices. This is consistent with literature on habit theory which highlights the enabling effect of new contexts on new behaviours (Nilsen et al. [2008]).

There may be study limitations which diminish the external validity of our findings. Although we purposively sampled to achieve a balance of views by recruiting participants who consistently used the online surveillance system and those who did not, all interviewees had prior knowledge of, and possibly an interest in injury surveillance and their views may not be representative of the broader population of sports trainers. Also, our sample size was relatively small (n = 12) although, despite this, we did achieve saturation of content amongst our interviewees. Finally, we conducted this study within a sport that is relatively well-organised, in terms of sports trainer staffing. In other sports, contextual barriers, such as a lack of resources and staff, could be more significant than they were within Australian football, making some of the recommendations harder to implement and the findings less generalisable.

Based on our findings, there are a number of practical suggestions to improve the implementation of injury surveillance systems in similar settings. The voluntary nature of injury surveillance within community sport heightens the influence of an intrinsic belief in the importance of the practice. To increase understanding of the importance of injury surveillance, all club personnel should be educated on the importance of recording injuries for legal, communication and injury prevention (and consequently athlete and team performance) purposes. To avoid injury underreporting, athletes and coaches should be educated on the importance of reporting injuries and on taking the necessary steps to ensure injuries are rehabilitated properly before returning to play. Underreporting may also be reduced by providing athletes and coaches with a standardised definition of a reportable injury. Based on our finding of greater implementation of the injury surveillance system among clubs receiving full support for the delivery of FootyFirst, it would appear that there is great benefit in engaging with coaches, club presidents and league officials about injury surveillance. Therefore, sporting leagues should support injury surveillance practices and create clear guidelines as to their expectations. Clubs, leagues and governing bodies should also appreciate the workload of sports trainers and seek to increase staffing levels and remuneration for these individuals. Finally, online tools can be made more accessible by developing smartphone and tablet applications and by reducing technical issues. Additionally, acknowledging that not all users will easily adapt to new technology, there is a need to be flexible in offering different modes of surveillance, including paper-based forms.

## Conclusions

This research offers important insights into the factors affecting the implementation of injury surveillance systems in community sports settings. We achieved a relatively low level of implementation and maintenance following delivery of our implementation strategy. However, results were best in those leagues that were receiving concurrent delivery support for an injury prevention program, suggesting that greater levels of engagement with staff at all levels and with relevant organisations can enhance uptake. Personal factors, such as a belief in the importance of injury surveillance were influential, as were socio-contextual factors, such as athlete underreporting and understaffing. Finally, to increase reach, adoption, implementation and maintenance, surveillance systems should be user-friendly and delivered in a range of accessible formats. The findings of this research should be considered when attempting to improve the uptake of injury surveillance systems in sports and other settings where surveillance is optional. By increasing uptake of surveillance systems, surveillance data will represent a greater proportion of the target population and increase our understanding of the extent of the injury problem in specific settings.

## Additional file

## Electronic supplementary material

Additional file 1: **Injury Incident form.** Sports Injury Tracker form used for recording injury details (Sports Medicine Australia [2012]). (PDF 127 KB)

Below are the links to the authors’ original submitted files for images.

Authors’ original file for figure 1

Authors’ original file for figure 2

## Abbreviations

SMA: Sports medicine Australia
NoGAPS: National guidance for Australian football partnerships and safety
CEO: Chief executive officer
AFL: Australian football league
RE-AIM: Reach, efficacy, adoption, implementation, maintenance
