# Supplementary material for: Implementing injury surveillance systems alongside injury prevention programs: evaluation of an online surveillance system in a community setting
Source: Inj Epidemiol. 2014 Jul 24;1(1):19. doi: 10.1186/s40621-014-0019-y (PMC4648950; doi:10.1186/s40621-014-0019-y)
Supplement: Supplementary file 1 — Additional file 1: Injury Incident form. Sports Injury Tracker form used for recording injury details (Sports Medicine Australia [2012]). (PDF 127 KB) [file 40621_2014_19_MOESM1_ESM.pdf]

SPORTSINJURYTRACKER

www.sportsinjurytracker.com.au

Name of patient: ______________________ DOB __/___/___ Date of Injury: __/__/__

Sex: Male ☐ Female ☐ Time ______:______am/pm Sport______________________

The injured person is: Player_Referee_Coach_Spectator Patient Adddress: ____Postcode ________

Patient phone number: ______________________ Venue_______________________________________ Event/match_____________________________

Type of activity at time of injury
☐ training
☐ warm-up
☐ competition
☐ cool-down
☐ other ______________________

Reason for Presentation
☐ new injury
☐ exacerbated/aggravated injury
☐ recurrent injury
☐ illness
☐ other ______________________

Body Region Injured
Tick or circle body part/s injured & name

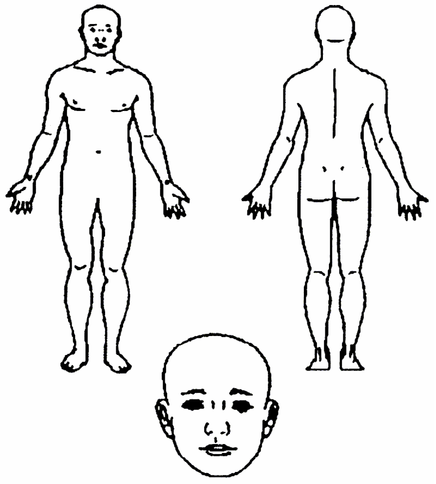

Body part/s

____

____

Nature of Injury/Illness
☐ abrasion/graze
☐ sprain eg ligament tear
☐ strain eg muscle tear
☐ open wound/laceration/cut
☐ bruise/contusion
☐ inflammation/swelling
☐ fracture (including suspected)
☐ dislocation/subluxation
☐ overuse injury to muscle or tendon
☐ blisters
☐ concussion
☐ cardiac problem
☐ respiratory problem
☐ loss of consciousness
☐ unspecified medical condition
☐ other ______________________

Provisional diagnosis/es

____

Mechanism of Injury
☐ struck by other player
☐ struck by ball or object
☐ collision with other player/referee
☐ collision with fixed object
☐ fall/stumble on same level
☐ jumping to shoot or defend
☐ fall from height/awkward landing
☐ overexertion (eg muscle tear)
☐ overuse
☐ slip/trip
☐ temperature related eg heat stress
☐ other ______________________

Explain exactly how the incident occurred: ______________________

____

____

Were there any contributing factors to the incident, unsuitable footwear, playing surface, equipment, foul play?

____

____

Protective Equipment
Was protective equipment worn on the injured body part? ☐ yes ☐ no

If yes, what type eg mouthguard, ankle brace, ______________________

Initial Treatment
☐ none given (not required)
☐ RICER ☐ dressing
☐ sling, splint ☐ crutches
☐ CPR ☐ stretch/exercises
☐ taping only
☐ none given - referred elsewhere
☐ other ______________________

Advice Given
☐ Immediate return, unrestricted activity
☐ Able to return with restriction
☐ Unable to return at the present time
☐ Able to return but the player chose not to
☐ Referred for further assessment before returning to activity

Referral
☐ no referral
☐ medical practitioner
☐ physiotherapist
☐ ambulance transport
☐ hospital
☐ other

____

Provisional severity assessment
☐ mild (1-7 days modified activity)
☐ moderate (8-21 days modified activity)
☐ severe (>21 days modified or lost)

Treating person
☐ medical practitioner
☐ sports trainer (ID ____________)
☐ other ______________________

☐ I have provided the patient with a copy of this report. I told the patient that this record will be kept for insurance purposes. The injury information will be entered into the Sports Injury Tracker Tool to monitor injuries that occur in sport to help to create a safer environment for the future.

Treating Persons Name

____

Signature ______________________

For more information about Sports Injury Tracker call 03 9674 8777
