# Supplementary figures and images for: Implementing injury surveillance systems alongside injury prevention programs: evaluation of an online surveillance system in a community setting
Source: Inj Epidemiol. 2014 Jul 24;1(1):19. doi: 10.1186/s40621-014-0019-y (PMC4648950; doi:10.1186/s40621-014-0019-y)

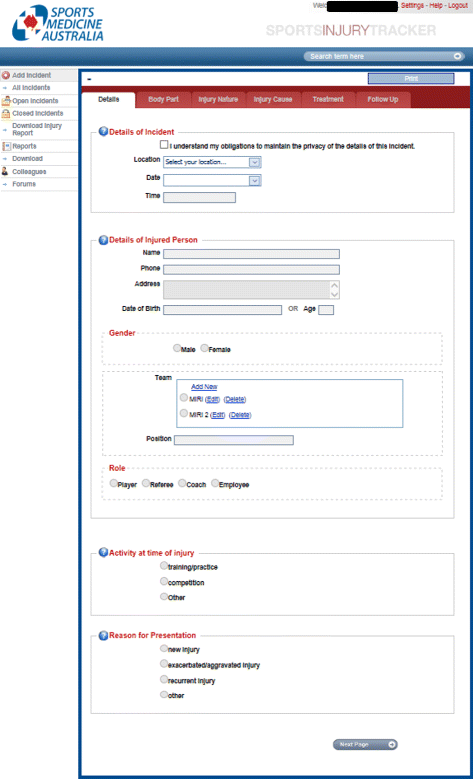

Supplement: Supplementary file 2 — Authors’ original file for figure 1 [file 40621_2014_19_MOESM2_ESM.gif]

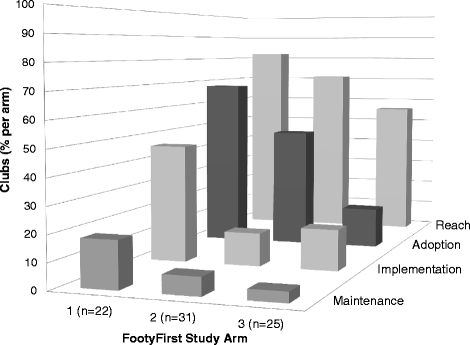

Supplement: Supplementary file 3 — Authors’ original file for figure 2 [file 40621_2014_19_MOESM3_ESM.gif]
